# Decoding the Role of O-GlcNAcylation in Hepatocellular Carcinoma

**DOI:** 10.3390/biom14080908

**Published:** 2024-07-25

**Authors:** Xinyu Zhou, Sirui Hang, Qingqing Wang, Liu Xu, Peter Wang

**Affiliations:** 1 Department of Surgery, Zhejiang Chinese Medical University, Hangzhou 310053, China; zhouxinyu@zcmu.edu.cn (X.Z.); hangsirui@zcmu.edu.cn (S.H.); 2 Department of Hepatobiliary Surgery, The First Hospital of Jiaxing, Jiaxing 314051, China; 15067334898@163.com; 3 Department of Medicine, Zhejiang Zhongwei Medical Research Center, Hangzhou 310000, China

**Keywords:** OGA, OGT, HCC, O-GlcNAcylation, proliferation, immunotherapy

## Abstract

Post-translational modifications (PTMs) influence protein functionality by modulating protein stability, localization, and interactions with other molecules, thereby controlling various cellular processes. Common PTMs include phosphorylation, acetylation, ubiquitination, glycosylation, SUMOylation, methylation, sulfation, and nitrosylation. Among these modifications, O-GlcNAcylation has been shown to play a critical role in cancer development and progression, especially in hepatocellular carcinoma (HCC). This review outlines the role of O-GlcNAcylation in the development and progression of HCC. Moreover, we delve into the underlying mechanisms of O-GlcNAcylation in HCC and highlight compounds that target O-GlcNAc transferase (OGT) and O-GlcNAcase (OGA) to improve treatment outcomes. Understanding the role of O-GlcNAcylation in HCC will offer insights into potential therapeutic strategies targeting OGT and OGA, which could improve treatment for patients with HCC.

## 1. Introduction

Hepatocellular carcinoma (HCC) is a common type of liver cancer [1]. In the United States in 2024, the estimated number of new cancer cases is 41,630, and the estimated number of new deaths is 29,840 [2]. Moreover, in 2022, there were 865,269 new cases of liver cancer and 757,948 new deaths worldwide [3]. HCC typically develops in patients with cirrhosis caused by hepatitis B or C infection or alcohol-related liver disease [4]. The risk factors of HCC include chronic viral hepatitis, cirrhosis, alcohol consumption, and non-alcoholic fatty liver disease [5,6,7]. Patients with early-stage HCC have no symptoms, and patients with late-stage HCC present with abdominal pain, swelling, jaundice, fatigue, and weight loss. Ultrasound, computed tomography (CT) scans, and magnetic resonance imaging (MRIs) are commonly used for the diagnosis of HCC [8,9]. Blood tests, such as alpha-fetoprotein (AFP) and liver function tests, can be helpful for HCC diagnosis, while a biopsy can be performed to confirm the presence of HCC [10]. Early detection is important for a better prognosis of HCC because the prognosis varies depending mainly on the stage at the time of diagnosis. The 5-year relative survival rate for patients with liver cancer is 22%, suggesting that it is critical to develop approaches for early detection [2].

The treatments for HCC include liver resection, liver transplantation, ablative therapies, transarterial chemoembolization (TACE), targeted therapy, and immunotherapy [11]. The chemotherapeutic drugs used to treat HCC include doxorubicin (adriamycin), cisplatin, 5-fluorouracil, and gemcitabine [12]. These chemotherapeutic agents, which are highly toxic, have not shown substantial benefits in improving survival. Sorafenib, a targeted therapeutic agent, is a tyrosine kinase inhibitor that blocks tumor cell proliferation and angiogenesis. Other targeted therapy agents include lenvatinib, regorafenib, and cabozantinib [13]. Bevacizumab targets the VEGF pathway to block angiogenesis [14]. Immunotherapeutic agents include nivolumab (Opdivo), pembrolizumab (Keytruda), and atezolizumab (Tecentriq), and function as immune checkpoint inhibitors (ICIs) [15]. The common immune checkpoints in the immune system are programmed death-1 (PD-1) and programmed death-ligand 1 (PD-L1). These checkpoints are important for maintaining self-tolerance and for modulating the immune response during infections and inflammation [16]. PD-1 is a protein found on the surface of T cells, whereas PD-L1, a ligand of PD-1, is a protein expressed on the surfaces of many cells, including tumor cells, macrophages, and dendritic cells [17]. When PD-1 binds to PD-L1, it sends an inhibitory signal to T cells, leading to a reduction in T cell activity. This reduction in activity leads to escaping immune surveillance and growing unchecked [18]. ICIs that block PD-1 or PD-L1 disrupt the binding between PD-1 and PD-L1, resulting in increased immune response against cancer cells [19]. Nivolumab and pembrolizumab were developed for targeting the PD-1 receptor on T cells, whereas atezolizumab was developed for targeting the PD-L1 protein. Immunotherapy could revolutionize the treatment of HCC [20]. Early detection and treatment are crucial strategies for reducing the risk of developing HCC and improving patient prognosis.

## 2. O-GlcNAcylation

Post-translational modifications (PTMs) are chemical alterations of a protein after protein translation is complete. PTMs often affect protein function through the regulation of protein stability, localization, and interaction with other molecules [21]. PTMs have been reported to regulate various cellular processes. More than 400 different types of PTMs have been reported [22]. Some common types of PTMs have been identified, including phosphorylation, acetylation, ubiquitination, SUMOylation, methylation, glycosylation, sulfation, and nitrosylation [23]. Phosphorylation involves the addition of a phosphate group to serine, threonine, or tyrosine residues in proteins and is one of the most studied modifications in signaling pathways [24]. Acetylation involves the addition of an acetyl group to a lysine residue in a protein, which often regulates gene expression to affect histone modification and chromatin structure [25]. Ubiquitination involves the attachment of ubiquitin to lysine residues on a target protein, leading to protein degradation by the proteasome, which influences many cellular processes, such as cell cycle control, DNA repair, proliferation, and apoptosis [26,27]. SUMOylation is a type of PTM in which a small ubiquitin-like modifier (SUMO) is covalently attached to lysine residues in target proteins, which alters the function and localization of the modified proteins [28]. Methylation involves the addition of a methyl group to lysine or arginine residues in proteins [29]. Glycosylation involves the attachment of sugar molecules to lysine or asparagine residues in proteins, which is important for the function and localization of membrane-bound and secreted proteins [30]. Sulfation involves the addition of sulfate groups to tyrosine residues in proteins, which is critical in the modification of hormones and certain receptors [31]. Nitrosylation involves the addition of a nitric oxide group to cysteine residues in proteins, which can affect the activity and function of these proteins in response to nitric oxide [32].

**Figure 1:**
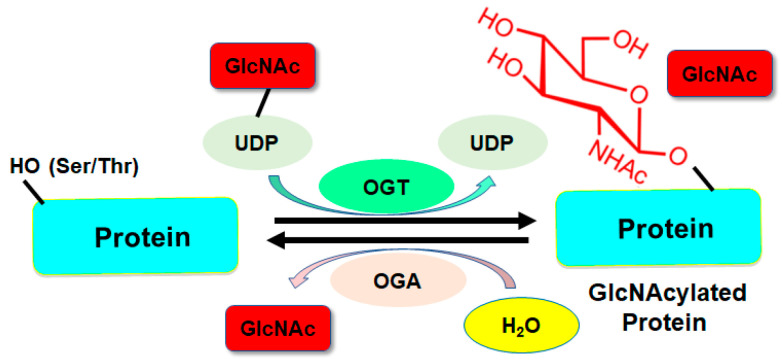
Illustration of O-GlcNAcylation. O-GlcNAcylation is a type of PTM in which a single N-acetylglucosamine (GlcNAc) sugar molecule is attached to serine or threonine residues in proteins. O-GlcNAcylation is regulated by O-GlcNAc transferase (OGT) and O-GlcNAcase (OGA). OGT adds the GlcNAc group to proteins, whereas OGA removes the GlcNAc group. The balance between OGT and OGA determines the O-GlcNAcylation status of proteins.

**Figure 2:**
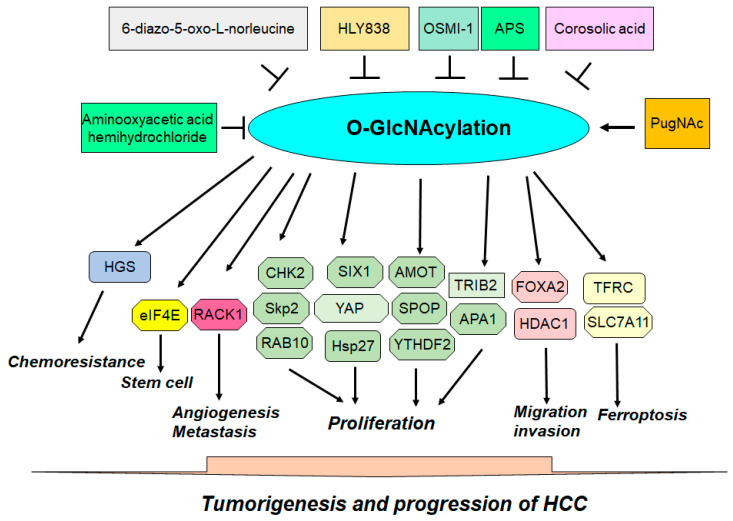
Role of O-GlcNAcylation in liver cancer. The O-GlcNAcylation of proteins plays a critical role in the development and progression of HCC. The inhibition of O-GlcNAcylation could be a potential approach for the treatment of patients with HCC whose O-GlcNAcylation is dysregulated.

Recently, O-GlcNAcylation has been shown to regulate tumorigenesis in various cancer types [33,34,35]. O-GlcNAcylation is a specific type of PTM in which a N-acetylglucosamine (GlcNAc) molecule is attached to serine or threonine residues in proteins [36,37]. Besides O-GlcNAcylation, cysteine S-linked N-acetylglucosamine (S-GlcNAcylation) is a PTM in mammals [38]. O-GlcNAcylation is dynamic and reversible and plays a crucial role in regulating cellular processes [39]. O-GlcNAcylation is controlled by two main enzymes: O-GlcNAc transferase (OGT) and O-GlcNAcase (OGA). OGT catalyzes the addition of O-GlcNAc to serine or threonine residues on target proteins, leading to O-GlcNAcylation. OGT transfers the GlcNAc moiety from the UDP-GlcNAc to specific hydroxyl groups on proteins. In general, OGT is often upregulated in cancers. OGA catalyzes the removal of O-GlcNAc groups from serine or threonine residues on proteins, leading to de-O-GlcNAcylation. OGA cleaves the β-glycosidic bond between GlcNAc and the hydroxyl group of the amino acid [40]. Hence, the balance between OGT and OGA determines the O-GlcNAcylation status of proteins (Figure 1). O-GlcNAcylation has been confirmed to regulate protein functions, the cell signaling pathway, and the stress response [41,42]. Abnormal O-GlcNAcylation has been reported to be associated with various diseases, including cancer, diabetes, cardiovascular disease, and Alzheimer’s disease [43,44,45,46]. O-GlcNAcylation has been verified to play essential roles in oncogenesis and tumor progression, including in HCC [47,48]. In this review, we describe the function of O-GlcNAcylation in HCC development and discuss the mechanism of O-GlcNAcylation in the development and progression of HCC. In addition, we describe compounds that target OGT and OGA to improve the treatment outcomes of patients with HCC. Understanding the role of O-GlcNAcylation in HCC could provide evidence for the development of potential therapeutic agents that target OGT and OGA for the treatment of patients with HCC (Figure 2).

## 3. Role of O-GlcNAcylation in HCC

Research has demonstrated that global O-GlcNAcylation levels are markedly increased in HCC tissues. Additionally, compared with those from patients without recurrence, tumor tissues from patients who experienced HCC recurrence after liver transplantation (LT) presented significantly increased O-GlcNAcylation levels [49]. Low OGA expression was identified as an independent prognostic factor for tumor recurrence in patients with HCC post-LT, particularly in those with low AFP levels. O-GlcNAcylation significantly influences cell viability, migration, and invasion in HCC by modulating the expression levels of E-cadherin, MMP1, MMP2, and MMP3 [49]. Numerous studies have revealed the substrates of O-GlcNAcylation in liver cancer and their underlying mechanisms in liver cancer development [47,50,51]. Hence, we describe the substrates of O-GlcNAcylation and their functions in liver cancer development and progression (Table 1 and Table 2).

## 4. O-GlcNAcylation of Hsp27

Hsp27 (heat shock protein 27), also known as HSPB1, is a small heat shock protein that plays crucial roles in several cellular processes [71]. In the context of cancer, Hsp27 is involved in promoting tumor development and progression and drug resistance [72,73]. The role of Hsp27 in HCC has been studied in recent years. Joo et al. reported that Hsp27 was expressed in 61.9% of patients with HCC and was associated with a subgroup of HBV-associated HCCs [74]. Luk et al. performed proteomic profiling analysis and reported that Hsp27 upregulation was associated with better prognosis in patients with HCC [75]. Guo et al. reported that Hsp27 could be involved in the regulation of apoptosis, migration, and invasion through the inactivation of NF-κB in HCC cells [76]. Cheng et al. uncovered that Hsp27 promoted colony formation and invasion in HCC cells partly via the regulation of multiple signaling pathways, such as the Wnt, ErbB, and TGF-beta signaling pathways [77]. Moreover, Hsp27 and annexin 1 (ANX1) were identified as biomarkers for invasive HCC and potential treatment targets [78].

Hsp27 was found to facilitate tumor metastasis through activation of the Akt signaling pathway in HCC, suggesting that the inhibition of Hsp27 can reduce the aggressiveness of HCC [79]. Furthermore, CD13-mediated autophagy enhanced chemoresistance via the p38/Hsp27/CREB/ATG7 axis in HCC [80]. The suppression of Hsp27 potentiated the sensitivity of hepatoma cells to 5-FU and carboplatin [81]. Similarly, Hsap27 expression was found to be associated with sensitivity to 17-allylamino-17-demethoxygeldanamycin in HCC cells [82]. Hsp27 has been reported to be regulated by PTMs. Ge et al. reported that SUMO2/3 promoted the SUMOylation of Hsp27, leading to the promotion of cell proliferation and invasive activity in HCC [83]. Hsp27 underwent O-GlcNAc glycosylation in HCC cells. This, the O-GlcNAcylation of Hsp27, potentially represents a new regulatory mechanism for Hsp27 function in HCC cells, especially regarding its nuclear translocation. The interaction between O-GlcNAcylation and Hsp27 phosphorylation may influence its subcellular distribution and biological activities in HCC [52].

## 5. O-GlcNAcylation of HDAC1

Histone deacetylase-1 (HDAC1) is an enzyme that regulates gene expression by modifying the acetylation status of histones and nonhistone proteins. HDAC1 is involved in chromatin remodeling and transcriptional regulation. HDAC1 removes acetyl groups from lysine residues in both histone and nonhistone proteins. The histone acetylation state influences how tightly or loosely DNA is wrapped around histones. By deacetylating histones, HDAC1 leads to a more condensed chromatin structure, which is generally associated with transcriptional repression. By deacetylating nonhistone proteins, HDAC1 influences various cellular processes in addition to transcriptional regulation. Aberrant HDAC1 activity has been linked to various diseases, especially cancer. The overexpression or dysregulation of HDAC1 is observed in many cancers, including HCC, and is associated with its prognosis [84,85]. One study revealed that the dysregulation of HDAC1 in HCC and its epigenetic influence on the transcription of genes related to autophagy and cell cycle components were significant. The overexpression of HDAC1 could be critical because it systematically regulates mitotic effectors involved in HCC progression, making it a particularly promising target for cancer therapy [86]. Pharmacological targeting of proteins that interact with HDAC1/3 led to morphological alterations and inhibited the proliferation and migration of HCC cells [87]. HDAC1 was found to be highly expressed in HCC, and two primary sites of O-GlcNAcylation were identified within its deacetylase domain. O-GlcNAcylation enhanced the phosphorylation and enzymatic activity of HDAC1. Functional analysis revealed that O-GlcNAc-modified HDAC1 mutants influenced the transcriptional regulation of p21 by altering histone acetylation levels, thereby impacting HCC cell proliferation. Additionally, mutations at the O-GlcNAcylation sites of HDAC1 affected the invasion and migration capabilities of HCC cells. These findings highlight the potential of OGT-mediated HDAC1 O-GlcNAcylation as a facilitator of HCC development, suggesting that targeting this modification could be a viable strategy to inhibit HCC progression [53].

## 6. O-GlcNAcylation of TFRC

Ferroptosis is a kind of programmed cell death that is characterized primarily by the accumulation of lipid peroxides to lethal levels in cells. The process is iron-dependent because iron plays a crucial role in driving the oxidation of lipids, which ultimately causes the cell to die [88]. Transferrin receptor (TFRC), also known as cluster of differentiation 71 (CD71), is a cell membrane receptor that is essential for iron uptake in cells. After TFRC binds to transferrin, cells use receptor-mediated endocytosis and internalize the transferrin–iron complex. Transferrin releases iron for multiple cellular processes. High levels of TFRC can lead to increased iron uptake and, consequently, increased susceptibility to lipid peroxidation and ferroptosis. Dysregulation of TFRC has been reported to be involved in the development of various cancers, including HCC. One study showed that TFRC and FTH1 were silenced by estrogen in liver cancer, leading to reductions in cell growth and survival [89]. TRIB2 regulated the β-TrCP-induced ubiquitination of TFRC, resulting in ferroptosis desensitization in liver cancer cells [90].

TFRC, HRAS, and MAPK3 were found to be correlated with ferroptosis and the tumor immune microenvironment in HCC [91]. Similarly, another study confirmed that TFRC was linked to sensitivity to ferroptosis inducers, such as lenvatinib, sorafenib, and artesunate, in HCC [92]. TFRC1 facilitated tumor metastasis and progression through activation of the mTOR pathway in HCC [93]. TFRC was modified by O-GlcNAcylation, affecting its sensitivity to erastin-induced ferroptosis in liver cancer cells. Further detailed investigations revealed that erastin prompted the removal of O-GlcNAcylation from TFRC at Ser687. This change reduced its interaction with the ubiquitin E3 ligase MARCH8, leading to decreased polyubiquitination at Lys665. As a result, the stability of TFRC increased, promoting the accumulation of labile iron [54]. O-GlcNAcylation increased the susceptibility of HCC cells to ferroptosis through the action of YAP. Additionally, YAP enhanced iron levels by increasing the transcription of TFRC, which was facilitated by its O-GlcNAcylation in HCC cells. When YAP was knocked down, the increased sensitivity to ferroptosis triggered by O-GlcNAcylation was abrogated. Furthermore, O-GlcNAcylation promoted ferroptosis sensitivity through TFRC in mice. These findings indicate that O-GlcNAcylation enhances ferroptosis sensitivity via TRFC in HCC cells [55].

## 7. O-GlcNAcylation of YAP

The Hippo signaling pathway is crucial for controlling organ size, tissue regeneration, and cell proliferation. The Hippo pathway has a kinase cascade: MST1, MST2 (mammalian Ste20-like kinases), LATS1, and LATS2 (large tumor suppressor kinases). LATS1 and LATS2 are activated by MST1/2, leading to the phosphorylation of YAP (Yes-associated protein) and TAZ (transcriptional coactivator with PDZ-binding motif). The phosphorylated YAP and TAZ are retained in the cytoplasm, while the dephosphorylation of YAP and TAZ results in their translocation to the nucleus and interaction with transcription factors to enhance the expression levels of genes that regulate cell proliferation. Dysregulation of the Hippo pathway has been implicated in liver cancer [94]. Overactive YAP or TAZ can lead to excessive cell growth and oncogenesis in liver cancer [95,96]. ITGAV was found to govern cell invasion via the regulation of YAP and TAZ in liver cancer cells [97]. The TNFR2/hnRNPK axis facilitated tumor development in liver cancer through the upregulation of YAP in hepatic progenitor cells [98]. YAP induced UHMK1 and enhanced the nuclear enrichment of MYBL2, leading to the promotion of cell proliferation in liver cancer cells [99].

PDE4D (phosphodiesterase 4D), a cAMP-hydrolyzing enzyme, has been reported to bind and interact with YAP to enhance tumor progression in HCC [100]. O-GlcNAcylation increased the expression, stability, and functionality of YAP in liver cancer. A specific O-GlcNAc modification site was found on YAP at Thr241, and alteration of this site led to the reduced O-GlcNAcylation, decreased stability, and diminished protumorigenic activities of YAP while enhancing its phosphorylation. Crucially, the O-GlcNAcylation of YAP was necessary for liver cancer development induced by high glucose levels. Additionally, a reciprocal relationship between YAP and overall cellular O-GlcNAcylation was discovered [56]. Interestingly, chlorogenic acid (CA) has been reported to reduce the expression of YAP and O-GlcNAcylation by suppressing the activity of CDK19. In addition, chlorogenic acid inhibited the activation of the HBP and decreased the expression levels of OGT and YAP under high-glucose conditions. Chlorogenic acid was found to reduce cell proliferation by attenuation of the CDK19/YAP/O-GlcNAcylation pathway in liver cancer, indicating that chlorogenic acid could serve as a compound for treating diabetes-associated liver cancer [101].

## 8. O-GlcNAcylation of AMOT

AMOT (angiomotin) is a crucial protein that interacts with components of the Hippo signaling pathway, particularly those that play roles in the regulation of YAP and TAZ [102,103]. AMOT is known to be involved in controlling cell polarity, migration, and angiogenesis [104]. AMOT has been reported to mediate cell proliferation in liver cancer. For example, the p130 isoform of AMOT is pivotal for YAP-induced tumorigenesis and cell proliferation in hepatic epithelial cells [105]. Zhu et al. reported that TPA (12-O-tetradecanoylphorbol-13-acetate) inhibited YAP translocation via AMOT and repressed tumor growth in liver cancer [106]. Liu et al. reported that AMOT can undergo O-GlcNAcylation. High glucose levels were found to increase the O-GlcNAcylation of AMOT. Under normal glucose conditions, AMOT functioned as an inhibitor of YAP, whereas HG facilitated the nuclear accumulation of YAP through AMOT. Thus, targeting AMOT O-GlcNAcylation might offer a more effective therapeutic strategy for treating liver cancer, particularly when associated with diabetes [57].

## 9. O-GlcNAcylation of TRIB2

Tribbles pseudokinase 2 (TRIB2) can mediate the degradation of target proteins [107]. TRIB2 structurally resembles kinases and shares similar domains, but it lacks certain key residues required for enzymatic activity. Instead of functioning as a true kinase, TRIB2 acts as a molecular scaffold or adaptor in various signaling pathways, influencing the regulation of protein stability and localization. TRIB2 has been shown to be involved in tumorigenesis, therapy resistance, and stem cell fate decisions [108,109]. One study revealed that TRIB2 is a downstream target of the Wnt pathway and regulates YAP and C/EBPα in liver cancer cells [110]. Another study reported that the dysregulation of phosphorylation and ubiquitination by p70S6K and Smurf1 led to the stability of TRIB2 and an oncogenic phenotype in liver cancer [111]. Qiao et al. reported that β-TrCP controlled the stability of TRIB2 in liver cancer cells [112]. Xu and coworkers reported that TRIB2 suppressed the Wnt/TCF4 pathway through several E3 ligases, including β-TrCP, COP1, and Smurf1, in liver cancer cells [113]. Moreover, TRIB2 regulated proteasome function to inhibit ubiquitin stability, resulting in the protection of liver cancer cells against oxidative stress [114]. The O-GlcNAcylation of TRIB2 induced by the HBP increased its protein stability, which in turn promoted transformative characteristics in liver cancer cells. Additionally, TRIB2 stabilized GUCY1A3 (guanylate cyclase 1 alpha 3) by interacting with it and reducing its ubiquitination [58].

## 10. O-GlcNAcylation of HGS

HGS (hepatocyte growth factor-regulated tyrosine kinase substrate), also known as HRS, is a critical ESCRT (endosomal sorting complex required for transport) component that plays crucial roles, particularly in the regulation of endocytosis and trafficking of the receptor [115,116]. One study suggested that HGS was highly expressed in pituitary adenoma [117]. Another study revealed that TP53 signaling affected HGS-induced exosome formation in colorectal cancer [118]. In addition, Beclin 1 enhanced the endosomal recruitment of HRS and inhibited tumor proliferation [119]. HRS influenced the secretion of PD-L1 in small extracellular vesicles and was linked to the effectiveness of anti-PD-1 therapy [120]. Zhang et al. reported that disrupting HRS suppressed tumor growth by enhancing CD8+ T-cell infiltration in melanoma and colon cancer. The absence of HRS caused the accumulation of misfolded proteins and induced endoplasmic reticulum stress, activating type I interferons through IRE1α and XBP1. HRS was found to be elevated in tumor cells characterized by a high TMB. HRS expression correlated with the effectiveness of PD-L1/PD-1 blockade therapy in melanoma. Additionally, HRS deletion increased the sensitivity of mice with melanoma to anti-PD-1 treatment [121]. Depletion of the vesicular sorting protein HRS suppressed tumor growth and metastasis through the regulation of β-catenin and E-cadherin [122]. In liver cancer, crosstalk between HGS and β-catenin was detected. The depletion of HGS reduced cell growth and induced apoptosis in liver cancer cells via β-catenin [123]. The O-GlcNAcylation of HGS reduced its interaction with STAM (signal-transducing adaptor molecule), thus disrupting ESCRT-0 complex formation. Additionally, O-GlcNAcylation promoted the ubiquitination of HGS and decreased its protein stability. As a result, the O-GlcNAcylation of HGS impeded the sorting of EGFR into intraluminal vesicles and its subsequent degradation in lysosomes, contributing to EGFR accumulation. Moreover, the O-GlcNAcylation of HGS has been shown to enhance tumor growth in mice and to increase chemoresistance in liver cancer cells [59].

## 11. O-GlcNAcylation of CHK2

CHK2 (checkpoint kinase 2), which acts as a protein kinase, plays a critical role in the cellular response to DNA damage [124]. When DNA damage occurs, CHK2 is activated, which helps prevent the cell from entering mitosis until repairs are made [125]. CHK2 activation is typically triggered by ATM kinase, which senses DNA double-strand breaks. Once activated, CHK2 phosphorylates downstream targets, leading to cell cycle arrest, DNA repair, and apoptosis [126]. Owing to its function in DNA repair, CHK2 is considered a critical player in cancer development [127]. CHK2 governs cell metabolism in liver cancer through the promotion of SDH (succinate dehydrogenase) activity via the TCA (tricarboxylic acid) cycle. Increased CHK2 mRNA in blood was detected in patients with HCC [128]. UCN-01 (7-hydroxystauroporine), a protein kinase inhibitor, induced cell cycle arrest through the regulation of p53/p21 and CHK2/CDC25C in human hepatoma cells and reduced tumor cell invasion through the inhibition of β-catenin phosphorylation [129]. Dihydromyricetin increased cell cycle arrest in the G2/M phase by targeting the CHK1/CHK2/CDC25X axis, leading to the suppression of cell proliferation in HCC [130]. The overexpression and mislocalization of CHK2 in mitotic structures can increase chromosomal instability and accelerate the progression of HCC [131].

Reticulon-3-induced activation of CHK2/p53 suppressed the development of HCC, but this effect was inhibited by the HBV [132]. CCNDBP1 (cyclin D1 binding protein 1) responded well to DNA damage in HCC cells through regulation of the ATM/CHK2 pathway [133]. CHK2 signaling, which can be regulated by c-Rel, has been shown to orchestrate the DNA damage response, thereby curtailing the development of HCC [134]. Zhao et al. reported that the miR-34a-5p-c/MYC/CHK1/CHK2 axis mitigated cancer stem cell-like traits and increased radiosensitivity by inhibiting the DNA damage response in HCC [135]. PCK1 (phosphoenolpyruvate carboxykinase 1), a gluconeogenic enzyme, has been reported to be downregulated in HCC. Knocking out PCK1 increased global O-GlcNAcylation levels after exposure to low glucose. The loss of PCK1 in hepatoma cells triggered metabolic reprogramming, which led to the accumulation of oxaloacetate and increased de novo synthesis of uridine triphosphate, aiding in the production of UDP-GlcNAc. Additionally, PCK1 deletion caused inactivation of the AMPK–GFAT1 axis, further facilitating the synthesis of UDP-GlcNAc and thus elevating O-GlcNAcylation. Reduced PCK1 expression promoted the O-GlcNAcylation of CHK2 at threonine 378, which in turn increased CHK2-dependent Rb phosphorylation and accelerated cell proliferation. Furthermore, HBP-mediated CHK2 O-GlcNAcylation was inhibited by two compounds, aminooxyacetic acid hemihydrochloride and 6-diazo-5-oxo-L-norleucine, resulting in the suppression of tumor growth in liver-specific Pck1-knockout mice. Therefore, inhibiting the O-GlcNAcylation of CHK2 could be useful for treating HCC [60].

## 12. O-GlcNAcylation of APA1

Gap junction protein gamma 1 (GJC1), also known as connexin 45 (Cx45), a member of the connexin family of proteins, is a key component of gap junctions [136]. This communication is important for maintaining tissue and organ function. GJC1 is involved in the formation of gap junction channels, which facilitate the intercellular exchange of signaling molecules. Mutations or dysregulation of GJC1 can lead to several health issues, including cardiac abnormalities, developmental disorders, and cancer [137,138,139]. One study revealed through DNA methylation analysis that GJC1 was silenced by promoter hypermethylation in colorectal cancer [140]. Moreover, GJC1 promoter hypermethylation was validated in benign and malignant colorectal tumors. GJC1 methylation was correlated with BRAF mutations and proximal tumor location in colorectal cancer [141]. GJC1 was also confirmed to be a major component of gap junctions in HeLa cells [142]. GJC1 was identified as a proto-oncoprotein that can enhance the proliferative capacity of liver cancer cells under high-glucose conditions. The zinc finger protein APA1 underwent O-GlcNAcylation in liver cancer cells, a step that is critical for the HG-induced binding of APA1 to the GJC1 promoter. Compared with patients without diabetes, patients with both liver cancer and diabetes exhibited higher levels of global O-GlcNAcylation [61]. Furthermore, reducing O-GlcNAcylation eliminated the HG-driven increase in cell proliferation, which could be gradually restored by co-overexpressing APA1 and GJC1. Notably, patients with both liver cancer and diabetes presented significantly higher levels of global O-GlcNAcylation and expression of APA1 and GJC1 than did patients with just diabetes. Furthermore, reducing O-GlcNAcylation eliminated the HG-driven increase in cell proliferation, which could be gradually restored by the upregulation of APA1 and GJC1. Therefore, targeting GJC1 could be helpful for preventing diabetes-associated liver cancer [61].

## 13. O-GlcNAcylation of eIF4E

It has been accepted that eIF4E (eukaryotic translation initiation factor 4E) is a crucial component in the process of translation initiation in eukaryotic cells [143]. eIF4E is vital for the recruitment of mRNA to the ribosome, which is necessary for protein synthesis. Because it initiates protein synthesis, eIF4E is involved in cell growth, proliferation, and survival [144]. eIF4E is often found to be upregulated in various types of cancer, making it a target for therapeutic intervention [145]. One study revealed that eIF4E expression was upregulated in HCC tissues compared with nontumor tissues and was associated with tumor number, overall survival, and the disease-free survival rate. Hence, eIF4E expression could be a potential indicator for the survival of patients with HCC [146]. Cercosporamide suppressed eIF4E phosphorylation and led to the suppression of growth, survival, and angiogenesis in HCC [147]. The levels of total O-GlcNAcylation and OGT protein were elevated in HCC. OGT enhanced the stem-like cell features by activating eIF4E, which interacted with the 5′-UTR (untranslated region) of Sox2 in HCC. The O-GlcNAcylation of eIF4E was found to occur at Thr168 and THr177, leading to the protection of eIF4E from degradation. High glucose augmented stem-like cell function by targeting OGT and eIF4E in HCC. Overall, these findings indicate that OGT enhances stem-like cell traits by promoting the O-GlcNAcylation of eIF4E in HCC [62].

## 14. O-GlcNAcylation of SIX1

Sine oculis homeobox homolog 1 (SIX1) is a member of the SIX family of homeobox transcription factors, which play critical roles in several crucial processes, including muscle and kidney development and neurogenesis [148,149]. The aberrant expression or mutation of SIX1 has been linked to several disorders, including cancer [150]. Given its roles in cell growth and developmental pathways, SIX1 is considered a potential target for therapeutic intervention in cancer and developmental disorders [151]. Higher expression of SIX1 in both mRNA and protein levels was observed in HCC tissues than in nontumor liver samples. SIX1 protein overexpression was associated with TNM (tumor, node, metastasis), poor survival, and venous infiltration in HCC [152]. Downregulation of SIX1 by shRNA interference inhibited oncogenesis and metastasis in HCC [153]. One study revealed that HDAC5 upregulated the expression of SIX1 and promoted the proliferation of HCC cells [154]. Another group reported that SIX1 and DACH1 regulated the expression of p53 and governed cell apoptosis and proliferation in HCC [155].

Chu et al. observed that miR-204-5p inhibited cell proliferation through the regulation of SIX1 expression in HCC [156]. Tang and colleagues reported that the lncRNA CRNDE absorbed miR-337-3p and upregulated SIX1, leading to increased tumor progression in HCC [157]. The exosomal lncRNA TUG1 enhanced cell glycolysis, migration, and invasion by targeting the miR-524-5p/SIX1 pathway in HCC [158]. SIX1 was found to attenuate cancer stemness, was associated with poor prognosis, and reduced sensitivity to 5-FU in HCC [159]. Moreover, SNS-023 induced the degradation of SIX1 and RPS16, leading to sorafenib sensitivity in HCC [160]. In female patients with HCC who were HCV-positive, the upregulation of SIX1 was correlated with tumor growth and poor survival [161]. SIX1 was found to accelerate tumor cell growth in vitro and in vivo. Additionally, an increase in O-GlcNAcylation was observed, which was associated with SIX1. Crucially, O-GlcNAcylation substantially modified SIX1, preventing its breakdown through the ubiquitination pathway. Moreover, T276A SIX1 decreased O-GlcNAcylation levels, consequently reducing SIX1-mediated tumor-promoting capabilities. Hence, O-GlcNAcylation stabilized SIX1 and enhanced HCC cell proliferation. This study unveiled a unique interplay between SIX1 and O-GlcNAcylation, illustrating its vital role in bridging glucose metabolism and HCC progression [63].

## 15. O-GlcNAcylation of RACK1

RACK1 (ribosomal receptor for activated C-kinase 1) is a multifunctional scaffold protein that plays key roles in various cellular processes by mediating protein–protein interactions [162]. RACK1 belongs to the tryptophan–aspartate repeat (WD-repeat) family of proteins, which are involved in a multitude of cellular functions, including signal transduction, cell division, neural development, and apoptosis [163,164,165]. RACK1 is known to play pivotal roles in tumorigenesis and progression [166]. In HCC, RACK1 was found to promote tumor growth and chemoresistance through the promotion of eIF4E phosphorylation [167]. RACK1 regulated the IRE1/XBP1 pathway and interfered with sorafenib-mediated cell apoptosis in HCC [168]. Wang et al. reported that RACK1 increased the activity of JNK and promoted the proliferation of HCC cells [169]. RACK1 inhibited TNF-alpha-mediated ROS production and increased cell survival via CBR1 in HCC [170]. Similarly, one study showed that the depletion of RACK1 triggered cell apoptosis and attenuated cell proliferation in HCC cells [171].

Cao and coworkers reported that RACK1 stabilized Nanog activity and enhanced tumor cell self-renewal and chemoresistance in HCC [172]. In addition, RACK1 interacted with GNA14, which led to reductions in the MAPK/JNK and PI3K/AKT pathways, contributing to the inhibition of tumor progression in HCC [173]. Serine 122 in the RACK1 proteins was found to undergo substantial O-GlcNAcylation. This modification not only stabilized the RACK1 protein but also enhanced its association with ribosomes and its interaction with PKCβII (PRKCB), leading to the increased phosphorylation of eIF4E and the increased translation of key oncogenic proteins in HCC. Eliminating the O-GlcNAcylation of RACK1 at Ser122 markedly inhibited tumor growth, angiogenesis, and metastasis. Furthermore, the elevated O-GlcNAcylation of RACK1 was associated with tumor progression and increased recurrence rates following chemotherapy in patients with HCC [64]. Therefore, targeting the O-GlcNAcylation of RACK1 could be a potential strategy for HCC treatment.

## 16. O-GlcNAcylation of SLC7A11

SLC7A11 (solute carrier family 7 member 11) belongs to a specific subtype of transporters within the solute carrier family. The activity and expression of SLC7A11 are tightly regulated and can be influenced by various cellular conditions, including oxidative stress and the availability of growth factors [174]. Dysregulation of SLC7A11 is linked to several pathological conditions, including cancer [175,176]. SLC7A11 overexpression has been associated with increased cell survival, growth, and resistance to chemotherapy [177,178]. The RNA-binding protein DAZAP1 has been shown to interact with SLC7A11 mRNA and to govern ferroptosis, resulting in the promotion of HCC progression [179]. Similarly, circ0097009 regulated miR-1261/SLC7A11 and caused ferroptosis, subsequently influencing HCC progression [180]. Hypoxia inhibited METTL14-mediated silencing of SLC7A11 in a YTHDF2-dependent manner in HCC, leading to the inhibition of ferroptosis in HCC cells [181]. METTL9 was revealed to promote HCC progression via the inhibition of ferroptosis through SLC7A11 regulation [182]. Huang et al. reported that ABCC5 repressed SLC7A11-mediated ferroptosis and enhanced sorafenib resistance in HCC [183]. Li et al. reported that C8orf76 upregulated SLC7A11 expression and influenced ferroptosis in liver cancer cells [184]. Chen and colleagues observed that SOCS2 induced the ubiquitination of SLC7A11 and caused ferroptosis and increased radiosensitization in HCC [185].

The lncRNA HEPFAL governed the ubiquitination of SLC7A11 and accelerated ferroptosis in HCC [186]. Depletion of the lncRNA DUXAP8 increased sorafenib-mediated ferroptosis through the regulation of SLC7A11 depalmitoylation in HCC [187]. LncRNA CASC11 blocked sorafenib-mediated ferroptosis in HCC cells through SLC7A11 mRNA stabilization [188]. Similarly, LINC00942 regulated the IGF2BP3/SLC7A11 pathway, and suppressed ferroptosis and increased the immunosuppression of Treg cells in HCC [189]. lncRNA NRAV has been shown to regulate SLC7A11 via miR-375-3p and influence the prognosis of patients with HCC [190]. Aspirin was found to inhibit p65-induced SLC7A11 transcription and increase ferroptosis in HCC cells [191]. AKR1C3 was reported to target the YAP/SLC7A11 pathway and inhibit ferroptosis in HCC cells [192]. ATF4 inhibited hepatocarcinogenesis via SLC7A11 induction and suppressed stress-associated ferroptosis in HCC [193]. The depletion of SLC7A11 increased ferroptosis and attenuated tumor progression in HCC [194]. The inhibition of USP8 suppressed cell proliferation and increased ferroptosis in HCC and retarded tumor growth and lung metastasis in mice. The depletion of USP8 increased ROS accumulation in HCC cells. USP8 led to increased OGT stabilization via the inhibition of OGT ubiquitination. SLK-induced USP8 phosphorylation at S716 has been shown to be critical for binding with OGT. OGT led to SLC7A11 O-GlcNAcylation at Ser26 in HCC cells, promoting the import of cystine from the extracellular environment. Targeting USP8 suppressed SLC7A11 O-GlcNAcylation and enhanced ferroptosis through OGT upregulation in HCC [65].

## 17. O-GlcNAcylation of SPOP

SPOP (speckle-type POZ protein) is an adapter protein in the UPS that mediates protein degradation. Specifically, SPOP is a substrate adaptor of the Cullin-RING ligase complex (CRL3), where it functions as the substrate recognition subunit [195]. Mutations or alterations in SPOP are implicated in cancer [196,197]. For example, SPOP mutations have been frequently observed in prostate cancer and are linked to the pathogenesis of the disease [198]. Li et al. reported that CSN6/SPOP/HMGCS1 facilitated tumor progression via the activation of YAP1 in HCC [199]. BCLAF1 interacted with SPOP and led to the stabilization of PD-L1, resulting in the promotion of the development and immune escape of HCC [200]. SPOP was reported to abolish IRF2BP2-suppressed cell proliferation and metastasis in HCC [201]. In addition, SPOP increased the ubiquitination of CREB5 and inhibited the MET signaling pathway in liver cancer [202]. The SPOP protein underwent extensive O-GlcNAcylation by OGT at the Ser96 position in HCC. Typically, SPOP resides in the cytoplasm and facilitates the ubiquitination of Nogo-B. O-GlcNAcylation at Ser96 was shown to alter SPOP localization, moving it predominantly into the nucleus in hepatoma cells. This relocation reduced the ubiquitination and degradation of Nogo-B, subsequently promoting the progression of HCC. The ability of SPOP to target O-GlcNAcylation might underscore a potential strategy for HCC therapy [66].

## 18. O-GlcNAcylation of FOXA2

FOXA2 (forkhead box A2) is a transcription factor that belongs to the forkhead family of proteins and plays a critical role in the regulation of gene expression [203]. FOXA2 has been reported to take part in tumorigenesis in HCC. For example, miR-200a inhibited the expression of FOXA2 and reduced tumor metastasis and growth in HCC [204]. Silencing linc00261 induced tumor metastasis through the induction of FOXA2 transcription deficiency in HCC [205]. FOXA2 is O-GlcNAcylated by OGT, which influences the ability of HCC cells to migrate and invade. In HCC tissues, opposite expression patterns of FOXA2 and OGT were noted. Lower levels of FOXA2 were associated with poorer patient outcomes. The O-GlcNAcylation of FOXA2 led to the ubiquitin-dependent degradation of FOXA2, particularly in highly metastatic HCC cell lines. This modification of FOXA2 reduced the expression of E-cadherin, ultimately facilitating the migration and invasion of HCC cells. The O-GlcNAcylation of FOXA2 is critical for HCC metastasis [67].

## 19. O-GlcNAcylation of Skp2

Skp2 (S-phase kinase-associated protein 2) is an E3 ubiquitin ligase that is involved in cell cycle regulation. Skp2 has been reported to promote cell proliferation and tumor progression in HCC [206,207]. Skp2 downregulation inactivated Myc with HGF, resulting in the suppression of cell proliferation in liver cancer cells [208]. EAG1 modulated the expression of Skp2 and pseudopod formation and enhanced cell proliferation and metastasis in HCC [209]. The inhibition of SHIP2 by HBV X increased chemoresistance and tumor metastasis via Skp2 in HCC [210]. The potassium channel KCa3.1 activated the Skp2 and EMT pathways and promoted cell proliferation and metastasis in HCC [211]. Skp2 interacted with OGT and underwent extensive O-GlcNAcylation in HCC. This modification at Ser34 on Skp2 led to its stabilization by decreasing its degradation by the APC-CDH1 complex. Furthermore, O-GlcNAcylation enhanced the interaction of Skp2 with Skp1, improving its function as a ubiquitin ligase. This enhancement was shown to allow Skp2 to effectively facilitate the cell cycle G1–S phase transition by targeting p27 and p21 for degradation. Importantly, inhibiting the O-GlcNAcylation of Skp2 markedly reduced HCC cell proliferation [68].

## 20. O-GlcNAcylation of RAB10

RAB10 (Ras-related protein Rab-10) is a member of the RAB family of proteins, which belong to the larger RAS superfamily of small GTPases. RAB10 is crucial for regulating intracellular membrane trafficking [212]. Alterations in RAB GTPase expression have been linked to tumor cell proliferation, migration, and invasion in HCC [213]. The overexpression of RAB10 increased tumor growth and was associated with poor prognosis in HCC [214]. Zhang et al. reported that miR-519d triggered apoptosis and autophagy via activation of the AMPK pathway by RAB10 in HCC cells [215]. Cheng et al. revealed that miR-557 repressed tumor progression through the Wnt/β-catenin pathway by targeting RAB10 in HCC [216]. One study reported elevated levels of RAB10, OGT, and O-GlcNAcylation in HCC. Additionally, a significant positive correlation was observed between the protein levels of RAB10 and OGT expression. RAB10 was shown to directly bind with OGT in HCC cell lines, and this O-GlcNAcylation increased the stability of the RAB10 protein. Moreover, reducing OGT expression curtailed the aggressive characteristics of HCC, whereas increased RAB10 levels counteracted these effects. Hence, the O-GlcNAcylation of RAB10 induced by OGT enhances RAB10 stabilization, leading to accelerated HCC progression [69].

## 21. O-GlcNAcylation of YTHDF2

YTHDF2 (YTH domain-containing family protein 2) is a protein involved in the recognition and regulation of RNA modifications, particularly N6-methyladenosine (m6A) [217]. m6A is one of the most prevalent modifications found in mRNAs and regulates RNA stability, splicing, and translation. YTHDF2, as an m6A “reader”, binds to m6A-modified RNA and influences its fate [218]. YTHDF2 is associated with tumor progression and immune infiltration in HCC [219]. METTL3 (N6-methyladenosine methyltransferase-like 3) enhanced tumor progression via the YTHDF2-dependent inhibition of SOCS2 in HCC [220]. YTHDF2 inhibited cell growth and proliferation via the destabilization of EFGR mRNA in HCC [221]. One study showed that YTHDF2 inhibition facilitated vascular abnormalization and inflammation in HCC [222]. Another study revealed that YTHDF2 promoted cancer metastasis and increased cancer stemness by influencing OCT4 expression via m6A RNA methylation in liver cancer [223]. Liao et al. reported that Hsp90β impaired the STUB1-mediated ubiquitination of YTHDF2 and led to sorafenib resistance in HCC [224]. Recently, Wen et al. reported that YTHDF2 suppressed immune evasion and angiogenesis by targeting the ETV5/PD-L1/VEGFA pathway in HCC [225]. HBC infection increased YTHDF2 O-GlcNAcylation at Ser263 in HCC, which was induced by OGT. This modification increased the YTHDF2 stability and oncogenic function of YTHDF2 by suppressing its ubiquitination. Moreover, YTHDF2 increased the stability of the MCM2 and MCM5 transcripts, leading to the acceleration of cell cycle progression and HCC tumorigenesis. OSMI-1, an inhibitor of OGT, inhibited tumor progression in HCC [70].

## 22. O-GlcNAcylation and Immunotherapy

It has been documented that O-GlcNAcylation regulates tumor immunotherapy. For example, O-GlcNAc regulated the lysosomal degradation of PD-L1, and O-GlcNAc regulated HGS and blocked its interaction with PD-L1, contributing to impaired PD-L1 degradation. The suppression of O-GlcNAc led to the activation of T-cell-induced antitumor immunity. PD-L1 antibody plus O-GlcNAc suppression synergistically elevated the antitumor immune response. One inhibitor of HGS glycosylation reduced the PD-L1 expression and facilitated T-cell-mediated antitumor activity, suggesting that O-GlcNAc is involved in tumor immune evasion and immunotherapy [226]. One study showed that O-GlcNAc was linked to the HBP (hexosamine biosynthesis pathway). The silencing of OGT caused the activation of inmate immunity and increased septic inflammation. The OGT-driven O-GlcNAcylation of RIPK3 at threonine 467 inhibited the interactions between RIPK3 and RIPK1 and between RIPK3 molecules themselves, thereby blocking subsequent signaling for innate immunity and necroptosis. Hence, this modification has crucial immuno-metabolic interactions that are vital for regulating the activation of innate immune cells, underscoring the importance of glucose metabolism in managing septic inflammation [227]. Evidence has shown that MTHFD2 (methylenetetrahydrofolate dehydrogenase 2) can enhance basal and IFN-γ-induced PD-L1 expression. IFN-γ was shown to upregulate MTHFD2 through regulation of the AKT–mTORC1 axis. MTHFD2 stimulated the folate cycle to maintain adequate levels of uridine-associated metabolites such as UDP-GlcNAc, enhancing the global O-GlcNAcylation of proteins, such as c-Myc. This resulted in the increased stability of c-Myc and increased PD-L1 transcription. Correspondingly, a positive association was established between the levels of O-GlcNAcylation, MTHFD2, and PD-L1 in patients with pancreatic cancer [228]. Another group reported that elevated glucose flow through the HBP accelerated tumor progression and immune escape by augmenting O-GlcNAcylation in TAMs (tumor-associated macrophages). Increased O-GlcNAc levels shifted macrophage polarization toward an M2-like phenotype, leading to the promotion of tumor development. Additionally, elevated M2 markers on TAMs in patients with type 2 diabetes and colorectal cancer were observed [229]. In esophageal cancer, OGT enhanced cell growth and metastasis. OGT expression was increased in ALDH+ ECSCs (esophageal carcinoma stem cells). The depletion of OGT attenuated the self-renewal ability and tumorigenicity of ALDH+ ECSCs. OGT in exosomes upregulated PD-1 expression levels in CD8+ T cells, whereas OGT inhibition increased CD8+ T-cell-induced apoptosis in ALDH+ ECSCs. ECSCs can be protected by OGT in exosomes through PD-1 upregulation, leading to the promotion of cancer immunosuppression [230]. The role of O-GlcNAcylation in chemoresistance and radioresistance is still unclear. Therefore, it is necessary to determine whether OGT and O-GlcNAcylation regulate cancer immunosuppression, immunotherapy, chemotherapy, and radiotherapy in HCC.

## 23. Conclusions and Perspectives

In conclusion, the O-GlcNAcylation of proteins plays a pivotal role in the development and progression of HCC. The inhibition of OGT might be an alternative approach for treating HCC in which O-GlcNAcylation is dysregulated. Inhibitors of OGT could be useful as therapeutic agents for HCC treatment (Table 3). Understanding the regulatory mechanism of O-GlcNAcylation in HCC is important for the discovery of OGT inhibitors. Several compounds have been reported to target O-GlcNAcylation. For example, corosolic acid (CA) inhibited activation of the HBP and OGT expression and attenuated the expression of OGT, YAP, and O-GlcNAcylation through the suppression of CDK19. The upregulation of CDK19 abolished the corosolic acid-mediated inhibition of YAP and O-GlcNAcylation in HCC cells [101]. HLY838, a new diketopiperazine-derived OGT inhibitor, triggered a global reduction in the cellular O-GlcNAc level. HLY838 increased CDK9 inhibitor-mediated anticancer activity via the inhibition of c-Myc and E2F1 in HCC. At the molecular level, CDK9 controlled c-Myc expression at the transcriptional level, whereas OGT controlled CDK9 stabilization [231].

Astragalus polysaccharide (APS), obtained from Astragalus membranaceus, has been found to induce doxorubicin-mediated apoptosis and elevated endoplasmic reticulum stress. APS reduced the stability of OGT and induced OGA expression. PugNAc, an OGA inhibitor, abrogated the cell apoptosis and endoplasmic reticulum stress induced by doxorubicin and APS. Overall, APS reduced O-GlcNAcylation and increased doxorubicin-related apoptosis in HCC [232]. Several upstream factors have been reported to induce O-GlcNAcylation in HCC. Caveolin-1 (CAV1) increased the expression of OGT and O-GlcNAcylation in HCC cells, leading to increased cell migration [233].

It is required to mention that future perspectives for O-GlcNAcylation research are needed. Because O-GlcNAcylation has been linked to cancer, future research should focus on understanding how aberrations in O-GlcNAcylation contribute to carcinogenesis, which could result in novel therapeutic interventions. Studying the temporal and spatial dynamics of O-GlcNAcylation in cells will involve live-cell imaging and real-time analyses to explore how O-GlcNAcylation changes in response to various cellular states or external stimuli. It will be necessary to determine whether O-GlcNAcylation could be a potential biomarker for early diagnosis or cancer progression. These findings will be helpful for improving the early detection and treatment of cancer. To achieve this goal, the development of better analytical tools and techniques for the detection of O-GlcNAcylation is crucial. Moreover, it is pivotal to discover specific inhibitors of OGT using advanced techniques for patients with cancer whose O-GlcNAcylation is dysregulated. In addition, O-GlcNAcylation often involves interactions with other PTMs, such as phosphorylation, ubiquitination, and methylation. Future studies should explore these interactions between O-GlcNAcylation and other PTMs to uncover the complex regulatory networks involved in tumorigenesis. Given that O-GlcNAcylation is sensitive to the cellular metabolic state, particularly glucose levels, it will be necessary to explore how changes in diet, nutrition, and environmental factors influence O-GlcNAcylation and cancer. By addressing these questions, O-GlcNAcylation could be applied for cancer diagnosis and treatment.

## Figures and Tables

**Table 1:** Functions of O-GlcNAcylation in liver cancer.

Target	Mechanism	Function	Ref.
Hsp27	The O-GlcNAcylation of Hsp27 regulates its nuclear translocation.	The O-GlcNAcylation and phosphorylation of Hsp27 influence biological activities in HCC.	[52]
HDAC1	The O-GlcNAcylation enhances the phosphorylation and enzymatic activity of HDAC1 and influences the transcriptional regulation of p21 by altering histone acetylation levels.	O-GlcNAc-modified HDAC1 mutants impact HCC cell proliferation and cell invasion and migration capabilities in HCC.	[53]
TFRC	Erastin promotes the removal of O-GlcNAcylation from TFRC and reduces its interaction with MARCH8, leading to decreased polyubiquitination.	TFRC is modified by O-GlcNAcylation, affecting its sensitivity to Erastin-induced ferroptosis in HCC cells.	[54]
TFRC	The O-GlcNAcylation heightens the susceptibility of HCC cells to ferroptosis through the action of YAP.	O-GlcNAcylation promotes ferroptosis sensitivity through TFRC in HCC cells.	[55]
YAP	The O-GlcNAcylation increases the expression, stability, and functionality of YAP in liver cancer by regulating its phosphorylation.	The O-GlcNAcylation of YAP is necessary for liver cancer development induced by high glucose levels.	[56]
AMOT	High glucose levels increase O-GlcNAcylation of AMOT and facilitate the nuclear accumulation of YAP through AMOT.	Targeting AMOT O-GlcNAcylation offers an effective therapeutic strategy for treating liver cancer with diabetes.	[57]
TRIB2	The O-GlcNAcylation of TRIB2 increases its protein stability. TRIB2 stabilizes GUCY1A3 by interacting with it and reducing its ubiquitination.	The O-GlcNAcylation of TRIB2 promotes transformative characteristics in liver cancer cells.	[58]
HGS	The O-GlcNAcylation of HGS reduces its interaction with STAM, disrupts the ESCRT-0 complex, and promotes HGS ubiquitination and EGFR accumulation.	The O-GlcNAcylation of HGS enhances tumor growth in mice and increases chemoresistance in liver cancer cells.	[59]
CHK2	Reduced PCK1 expression promotes the O-GlcNAcylation of CHK2 and increases CHK2-dependent Rb phosphorylation.	The O-GlcNAcylation of CHK2 accelerates cell proliferation.	[60]

**Table 2:** Role of O-GlcNAcylation in liver cancer.

Target	Mechanism	Function	Ref.
APA1	APA1 undergoes O-GlcNAcylation, which is critical for the HG-induced binding of APA1 to the GJC1 promoter.	Reducing O-GlcNAcylation eliminates the HG-driven increase in cell proliferation.	[61]
eIF4E	The O-GlcNAcylation of eIF4E is located at Thr168 and THr177, resulting in the protection of eIF4E from degradation.	High glucose augments stem-like cell functions by promoting the O-GlcNAcylation of eIF4E.	[62]
SIX1	The O-GlcNAcylation of SIX1 prevents its breakdown through the ubiquitination pathway.	O-GlcNAcylation stabilizes SIX1 and enhances HCC cell proliferation.	[63]
RACK1	RACK1 O-GlcNAcylation stabilizes the RACK1 protein and enhances its association with ribosomes and PRKCB, leading to the elevated phosphorylation of eIF4E.	The O-GlcNAcylation of RACK1 increases tumor growth, angiogenesis, and metastasis.	[64]
SLC7A11	USP8 increases OGT stabilization by inhibiting OGT ubiquitination. OGT leads to SLC7A11 O-GlcNAcylation in HCC cells.	Promotes cystine importation from the extracellular environment and regulates ferroptosis.	[65]
SPOP	O-GlcNAcylation alters SPOP localization, moving it predominantly into the nucleus, reducing Nogo-B degradation.	This relocation enhances the progression of HCC.	[66]
FOXA2	The O-GlcNAcylation of FOXA2 leads to the ubiquitin-dependent degradation of FOXA2 in metastatic HCC cell lines.	The O-GlcNAcylation of FOXA2 is critical for HCC metastasis, invasion, and migration through E-cadherin inhibition.	[67].
Skp2	Skp2 O-GlcNAcylation causes its stabilization by decreasing its degradation by the APC-CDH1 complex and enhances its interaction with Skp1, improving its function.	Allows Skp2 to effectively facilitate the cell cycle G1–S phase transition by targeting p27 and p21 for degradation and induces HCC cell proliferation.	[68]
RAB10	RAB10 directly binds with OGT, and this O-GlcNAcylation increases the stability of the RAB10 protein.	Increased RAB10 levels promote the aggressive characteristics of HCC.	[69]
YTHDF2	HBC infection increases YTHDF2 O-GlcNAcylation, leading to increased YTHDF2 stability due to the suppression of its ubiquitination.	YTHDF2 enhances the stability of MCM2 and MCM5 transcripts and accelerates cell cycle progression and HCC tumorigenesis.	[70]

**Table 3:** Compounds target O-GlcNAcylation in HCC.

Compound	Target	Function	Ref.
OSMI-1	Inhibitor of OGT	Inhibits tumor progression in HCC.	[70]
Aminooxyacetic acid hemihydrochloride	HBP-mediated O-GlcNAcylation	Suppresses tumor growth in liver-specific Pck1-knockout mice.	[60]
6-diazo-5-oxo-L-norleucine	HBP-induced O-GlcNAcylation	Inhibits tumor growth in liver-specific Pck1-knockout mice.	[60]
Corosolic acid	OGT and HBP	Inhibits HBP activation and OGT expression and represses YAP and O-GlcNAcylation through CDK19 suppression.	[101]
HLY838	A new diketopiperazine-derived OGT inhibitor	Triggers a global reduction in cellular O-GlcNAc levels and increases CDK9 inhibitor-mediated anticancer activity through the inhibition of c-Myc and E2F1 in HCC.	[231]
APS	Reduces the stability of OGT and induces OGA expression	Induces doxorubicin-mediated apoptosis and elevated endoplasmic reticulum stress by reducing O-GlcNAcylation.	[232]
PugNAc	OGA inhibitor	Abrogates cell apoptosis and endoplasmic reticulum stress induced by doxorubicin and APS.	[232]
